# Overcoming Carrier Concentration Limits in Polycrystalline CdTe Thin Films with In Situ Doping

**DOI:** 10.1038/s41598-018-32746-y

**Published:** 2018-09-28

**Authors:** Brian E. McCandless, Wayne A. Buchanan, Christopher P. Thompson, Gowri Sriramagiri, Robert J. Lovelett, Joel Duenow, David Albin, Søren Jensen, Eric Colegrove, John Moseley, Helio Moutinho, Steve Harvey, Mowafak Al-Jassim, Wyatt K. Metzger

**Affiliations:** 1 Institute of Energy Conversion, University of Delaware, Newark, DE 19716 USA; 2 National Renewable Energy Laboratory, 15013 Denver West Parkway, Golden, CO 80401 USA

## Abstract

Thin film materials for photovoltaics such as cadmium telluride (CdTe), copper-indium diselenide-based chalcopyrites (CIGS), and lead iodide-based perovskites offer the potential of lower solar module capital costs and improved performance to microcrystalline silicon. However, for decades understanding and controlling hole and electron concentration in these polycrystalline films has been extremely challenging and limiting. Ionic bonding between constituent atoms often leads to tenacious intrinsic compensating defect chemistries that are difficult to control. Device modeling indicates that increasing CdTe hole density while retaining carrier lifetimes of several nanoseconds can increase solar cell efficiency to 25%. This paper describes *in-situ* Sb, As, and P doping and post-growth annealing that increases hole density from historic 10^14^ limits to 10^16^–10^17^ cm^−3^ levels without compromising lifetime in thin polycrystalline CdTe films, which opens paths to advance solar performance and achieve costs below conventional electricity sources.

## Introduction

Impurity doping to adjust the electron and hole concentrations over orders of magnitude in III-V and Si semiconductor materials has been understood for decades and has enabled numerous applications. However, the processing times to acquire sufficiently pure materials and large single crystals or grains limits obtainable costs and throughput. Thin films of highly absorptive materials such as CdTe, CIGS, CZTS, and perovskites can provide high throughput low material costs ideal for mass manufacturing of next-generation photovoltaics. However, it is very difficult to influence carrier concentration in these materials. The more ionic bonding in these materials contribute to strong self-compensation and stubborn defect chemistries that often lead to empiricism in lieu of clear scientific understanding and control.

Polycrystalline CdTe solar cells offer an example of where overcoming hole density limitations can lead to important technology shifts. Device modeling and experimental data shows that increasing doping from typical values of ~10^14^ cm^−3^ to >10^16^ cm^−3^ while retaining carrier lifetimes near 2 ns can increase the open circuit voltage (V_OC_) from mid-800 mV to >1 V, enable fill factors exceeding 85%, and push efficiency to 25%^1,2^. This would increase CdTe performance well beyond mc-Si, and reduce costs further below conventional energy sources. The historical CdTe doping limit is a symptom of defect compensation when relying on a mix of native point defects and poorly controlled impurities and doping mechanisms, which is a common issue with other polycrystalline compound semiconducting thin-film materials^3^. For example in CIGS, in which copper vacancies partly contribute acceptor defects, extrinsic additives such as Na, K, and O, appear to increase p-doping and reduce recombination, but the acceptor concentration is weakly correlated with dosage^4^. In CZTS, where native doping levels are much higher than CIGS, the challenge is controlling Zn-Cu stoichiometry to minimize exchange sites resulting in disorder that create sub-band gap defects^5^. In the above-mentioned polycrystalline materials, controlling the defect chemistry is the key to setting the doping level while reducing bulk and surface recombination.

Present-generation CdTe solar cells are based on a front wall superstrate configuration in which p-type CdTe or CdTe_1−x_Se_x_ alloy is deposited at rates of microns/min onto glass coated with transparent conducting films, which serve as the n-type junction partner, and possibly an n-type buffer layer such as CdS or MgZnO. In these cells, CdTe cell processing after growth relies on a series of treatments to supply Cl, O and Cu into the polycrystalline film stack, to fine-tune intra-grain stoichiometry, form acceptor complexes, and passivate surfaces^6^. Cells are completed by a back surface preparation to facilitate formation of a low-resistance electrical contact. Empirical refinement of this fabrication approach has produced CdTe films which repeatedly only reach mid-10^14^ cm^−3^ acceptor concentration. This low value will limit any future progress in performance, yet years of effort have failed to significantly increase acceptor density.

Doping CdTe single crystals has been demonstrated by incorporation of group I and group V elements^7,8^ in high temperature melts in Bridgman and traveling heating methods and recently by diffusion into polished wafers^9^. Similarly, epitaxially grown CdTe crystals with P or As have been made by molecular beam epitaxy and similar methods^10–12^. However, these methods are many orders of magnitude too expensive and too slow for photovoltaics. While it is possible to dope polycrystalline thin films by *ex-situ* diffusion, studies indicate that diffusion along grain boundaries is orders of magnitude greater than substitutional bulk diffusion, or the fast interstitial diffusion that occurs only with P^13,14^. This makes it challenging to obtain 10^16^ cm^−3^ doping with the uniformity, recombination rates, and processing temperatures currently required for practical solar technology.

In this paper we show that careful *in-situ* doping can be coupled with manufacturing methods to achieve uniform hole density exceeding 10^16^ cm^−3^. Vapor transport deposition (VTD) is currently being used to manufacture thin film solar cells. Here, a vapor transport deposition (VTD) process developed at the Institute of Energy Conversion^15^ was adapted to facilitate solid-state incorporation of group V elements into CdTe during growth by high temperature mixing of Cd, Te and group V dopant vapors prior to deposition. After growth of the dopant-incorporated CdTe films, rapid cooling (<10 sec) from deposition temperature to below 400 °C was performed to retain the high temperature state. As-deposited polycrystalline CdTe films, grown at rates of 10 µm/min at 20 Torr, exhibit hole density ranging from10^14^ cm^−3^ to 10^15^ cm^−3^ in films with 10^17^ cm^−3^ to 10^18^ cm^−3^ incorporation levels for three new dopants, P, As, and Sb. Activation of the dopants by short thermal annealing and subsequent quench cooling extends the hole density to >10^16^ cm^−3^.

## Results and Discussion

Obtaining p-type conductivity in CdTe relies on the nature of the defects used to create hole carriers. *Intrinsic* doping relies on the thermodynamic tendency of CdTe to form cadmium vacancies, *V*_*Cd*_, at elevated temperatures, where the single phase region widens asymmetrically on the Te side, and hence can accommodate Cd vacancies^16,17^. The *V*_*Cd*_ intrinsic defect forms an acceptor, and electron paramagnetic resonance estimates a transition energy within 0.47 eV above the valence band maximum^18,19^. Measured levels of intrinsically-formed acceptor concentration are often <10^16^ cm^−3^ in single crystal material^20^ which is substantially lower than expected from stoichiometric deviation, and higher levels are often not stable^21^. The problem is that while the desired *V*_*Cd*_ defect formation is favored, compensating antisite defects such as *Te*_*Cd*_ are also favored, leading to the neutral defect *V*_*Cd*_*-Te*_*Cd*_. Doping without impurities in CdTe is difficult, in fact, CdTe is often used in applications where very high resistivity is desired due to its strong tendency to compensate.

*Extrinsic* doping relies on activating point defects created by the incorporation of an impurity dopant. Standard CdTe solar cell processing generally introduces Cl and Cu. The Cl may occupy the Te site to form a donor, an interstitial site to form an acceptor, or a Cl_Te_-V_Cd_ acceptor complex. Similarly, Cu may occupy the Cd site to form an acceptor, an interstitial site to form a donor, or form complexes with intrsinic, Cl, and Cu defects. Studies have revealed how Cl and Cu defects can produce the strong compensation observed in practice, which will limit future technology advancement^22–24^.

In CdTe photovoltaics, a Group V doping approach on the anion site has received little attention until recently. Substitutional defects using P, As, and Sb on Te lattice sites form shallow acceptors with estimated transition energies for (0/-) of 0.07 eV, 0.10 eV, and 0.23 eV above the valence band maximum^21,25^, as listed in Table 1. In this scenario, the challenge is ensuring that both sufficient *V*_*Te*_ sites exist and that dopant substitution dominates over interstitial occupancy. In practice, annealing CdTe in the presence of dopants with a Cd overpressure has been shown to enable p-type doping levels >10^16^ cm^−3^ to be reached in bulk and epitaxial film single crystals using P and As^21,26,27^. First-principles investigations indicate comparable formation enthalpies for P, As and Sb substitutional defects in the CdTe lattice. However, at high Cd overpressure, the formation of Cd interstitials, *Cd*_*i*_, a deep donor or trap estimated at 330 meV below the conduction band may also form^28^. Furthermore, the difficulty in decomposing molecular phosphorous P_2_ and P_4_ species at the substrate together with the mismatch between the atomic radii of Te and P may complicate matters for P activation.

**Table 1:** Properties of group V dopants and corresponding substitutional defects.

Group V Dopant	pSat @550 C (Torr)	Covalent Radius (pm)	Defect	Energy Level *E*_*V*_ + (meV)	Defect Formation Enthalpy (eV)
P	>3000	106	**P**_**Te**_	50	1.83
As	220	120	**As**_**Te**_	100	1.68
Sb	0.012	140	**Sb**_**Te**_	230	1.72

Calculated defect transition energy levels and formation enthalpies from ref.^29^.

Adapting the VTD growth process for substitutional doping requires mitigating competing counter mechanisms to incorporate the dopants. High temperature growth, which produces high quality CdTe films with high electron lifetime thermodynamically favors formation of the intrinsic *V*_*Cd*_ defect, which could limit formation of the desired *V*_*Te*_ defect and lead to the compensating antisite defect *Te*_*Cd*_. However, the presence of the group V element in the growth environment shifts the equilibrium to allow the substitutional defect to form. To further push the system away from forming the *V*_*Cd*_ requires supplying excess Cd during the film growth followed by rapid cooling to retain the high temperature state. Thus to extend the VTD process for group V substitutional doping, the established VTD reactor modeling^15^ was modified to include mixing of the native Cd and Te vapors from subliming CdTe with excess Cd plus dopant vapors, to verify that a uniform effluent from the VTD source is achievable. An obstacle to dopant incorporation at high deposition temperature is the low sticking coefficient of P and As at the hot substrate surface. Here we incorporated: (1) vapor mixing to ensuring rapid growth to capture adsorbed species without promoting homogeneous vapor condensation; (2) rapid cool-down to ‘freeze-in’ a favorable high temperature defect stoichiometry and (3) establishment of a steady state condition in the source configuration throughout the deposition to provide lateral film composition uniformity.

**Figure 1:**
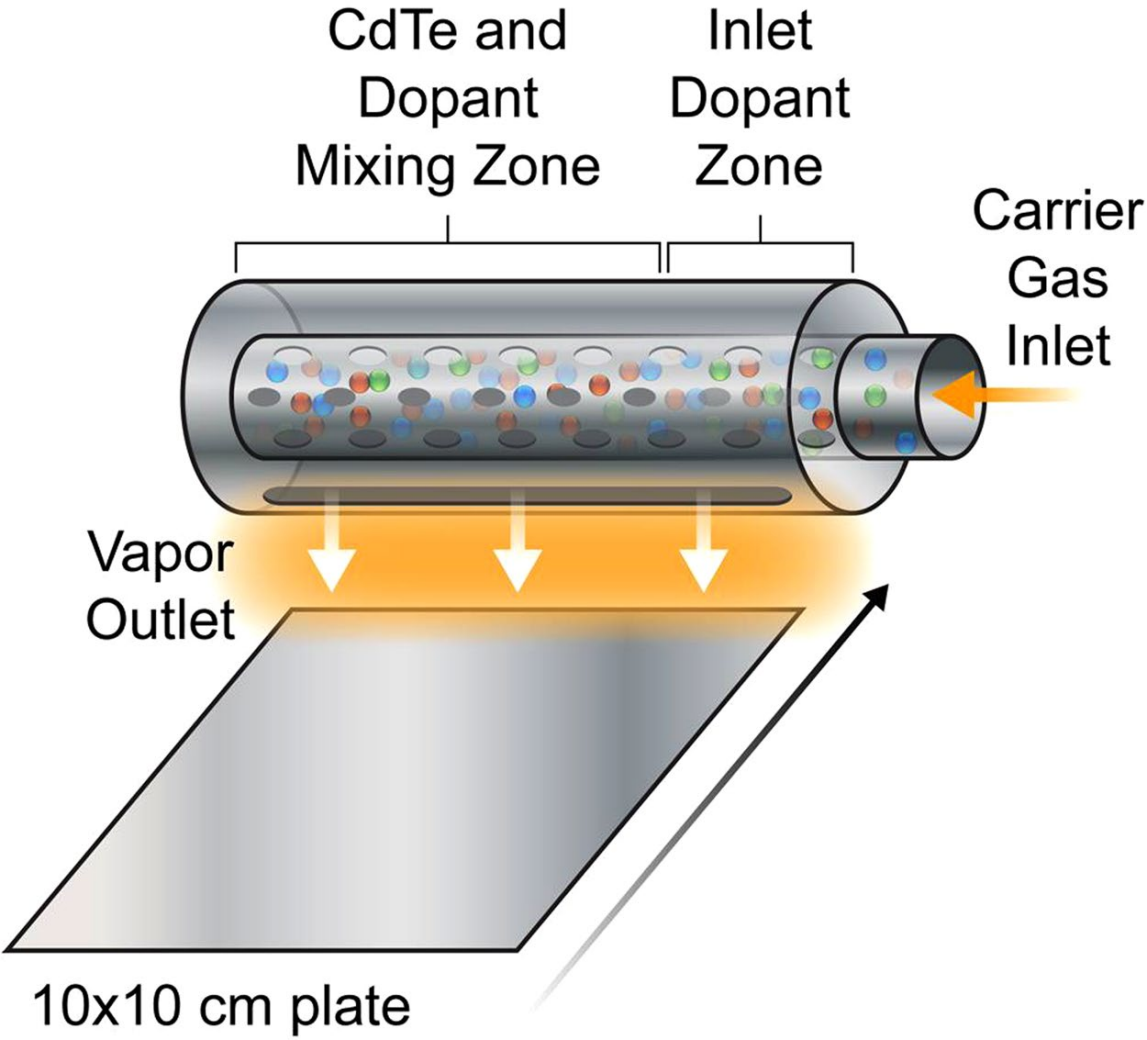
Schematic view of CdTe VTD deposition zone showing vapor source over translating substrate.

A VTD reactor was modified so that dopant and Cd excess vapor species could be entrained with the Cd and Te vapor generated from high purity CdTe source material as shown in Fig. 1. This *in-situ* entrainment of dopants in the CdTe film during deposition was achieved by addition of a high purity dopant-containing compound, specifically Cd_3_P_2_ for phosphorous, Cd_3_As_2_ for arsenic, and CdSb for antimony, along with elemental Cd, all located within the inlet zone of the CdTe source.

Sublimation of the four compounds, CdTe, Cd_3_P_2_, As_3_P_2_, and CdSb proceeds along the following^29^:

$${{\rm{CdTe}}}^{{\rm{s}}}\to {{\rm{Cd}}}^{{\rm{v}}}+0.5{{{\rm{Te}}}_{2}}^{{\rm{v}}}$$

$${{\rm{Cd}}}_{3}{{{\rm{P}}}_{2}}^{{\rm{s}}}\to 3{{\rm{Cd}}}^{{\rm{v}}}+0.5{{{\rm{P}}}_{4}}^{{\rm{v}}}\,{\rm{and}}\,0.5{{{\rm{P}}}_{4}}^{{\rm{v}}}\to {{{\rm{P}}}_{2}}^{{\rm{v}}}$$

$${{\rm{Cd}}}_{3}{{{\rm{As}}}_{2}}^{{\rm{s}}}\to 3{{\rm{Cd}}}^{{\rm{v}}}+0.5{{{\rm{As}}}_{4}}^{{\rm{v}}}\,{\rm{and}}\,0.5{{{\rm{As}}}_{4}}^{{\rm{v}}}\to {{{\rm{As}}}_{2}}^{{\rm{v}}}$$

$${{\rm{CdSb}}}^{{\rm{s}}}\to {{\rm{Cd}}}^{{\rm{v}}}+0.25{{{\rm{Sb}}}_{4}}^{{\rm{v}}}\,{\rm{and}}\,{{{\rm{Sb}}}_{4}}^{{\rm{v}}}\to 2{{{\rm{Sb}}}_{2}}^{{\rm{v}}}$$

where ‘s’ and ‘v’ denote solid and vapor species respectively. Within the source ampoule, operating at 840 °C, P exists primarily as the tetratomic molecule, Te, As and Sb exist in vapor as a binary molecule, and Cd exists in monatomic form. The concept developed is to utilize the spatial temperature profile within the source manifold to generate a vapor of dopant species and allow this to mix with the carrier gas containing Cd and Te_2_ vapor before exiting to the substrate, where supersaturation promotes condensation and film growth.

A mass transfer model of the VTD system was developed to estimate the concentrations of cadmium, tellurium, and dopant vapors effusing from the source. The model used the method of Hanket *et al*.^15^ with additional consideration of dopant species. First, we assume that mass transfer is governed by first-order saturation kinetics:

1 $$\frac{d{C}_{i}}{dx}=\frac{{C}_{i,eq}(T)-{C}_{i}}{v{\tau }_{D}(T)}$$

where *C*_*i*__(i = Cd, Te_2_, P_2_, P_4_, As_2_, As_4_, Sb) is the concentration of each gas phase species, *C*_*i*,*eq*_ is the concentration at equilibrium, T is absolute temperature, x is position along the enrichment zone, v is the velocity of the carrier gas, and *τ*_*D*_ is a characteristic diffusion time given as

2 $${\tau }_{D}=\frac{{l}_{D}^{2}}{D}$$

with *l*_*D*_ the diffusion length, taken as the diameter of the source enrichment ampoule, and *D* the diffusivity. Diffusivities were estimated using Chapman-Enskog theory^30^. The equilibrium concentrations of each species were calculated under the assumption that they are dilute with respect to the carrier gas. In the inlet dopant zone (see Fig. 1), Cd and dopant equilibrium concentrations were calculated using either experimental vapor pressure data or an activity coefficient model^29,31,32^. After the inlet zone, dopant concentration is constant and Cd and Te equilibrium concentrations are determined from vapor pressure data^29^.

The solution to Equation 1 gives an estimate of the concentration of gas phase species along the enrichment zone. Because velocity gradients enhance mass transfer, the first order model here should be taken as a lower limit on the gas phase species concentrations. The model showed that with placement of the compound dopant sources and Cd metal at the CdTe source ampoule inlet, positioned at the appropriate position on the thermal profile needed to achieve the desired temperature, adequate vapor mixing, with only 10% variation, could be achieved over the length of the source ampoule. For phosphorous doping using Cd_3_P_2_ at T_CdTe_ = 840 °C with T_Cd3P2_ = 450 °C, the concentration of phosphorous is 2 at%, corresponding to 8 × 10^20^ cm^−3^ if incorporated. For arsenic doping using Cd_3_As_2_ at T_CdTe_ = 840 °C with T_Cd3As2_ = 310 °C, the concentration of arsenic is 1 at%, corresponding to 4 × 10^20^ cm^−3^ if incorporated. For antimony doping using CdSb at T_CdTe_ = 840 °C with T_CdSb_ = 350 °C, the concentration of antimony is 1 at%, corresponding to 4 × 10^20^ cm^−3^ if incorporated. The emergent vapor flows axially into a boron nitride mixing baffle from which it flows downward out of the source exit slit onto the translating substrate.

The dopant position at the inlet of the deposition source was established using the model described above to achieve the calculated temperature required to produce a supplemental vapor stream concentration of ~10–100 ppm, which if fully incorporated, would yield targeted incorporation level ~10^18^ cm^−3^ atomic concentration in the CdTe film. We found that after each run, dopant material remained in the dopant zone of the VTD source after the run and was not depleted, indicating uniform delivery had proceeded throughout the run.

A variant method was also used employed in which the CdTe source was uniformly loaded with the dopant compound along its length, labeled the “uniform” source delivery method. For this, high purity CdTe powder was mechanically blended with the dopant compounds Cd_3_P_2_, Cd_3_As_2_, or CdSb, sealed in quartz ampoules, fired at 840 °C, and then rapidly cooled to room temperature by removal to a room temperature water bath. The resulting CdTe source material contained CdTe crystals with inclusions of dopant compounds, up to several weight percent. For this method to provide uniform dopant concentration over time, the mass loss rate from the sintered crystals in the source ampoule must be faster than the diffusion of the dopant from within each crystal to its surface, which would deplete the crystals of dopant, with a resulting decay in dopant incorporation over time. Based on measured mass loss, CdTe crystals within the source ampoule lose 1 micron/sec of CdTe. For P and As in CdTe, the characteristic diffusion length at 840 °C is 0.01 micron. Since the CdTe loss rate exceeds the dopant diffusion distance by 100X, the vapor concentration depends only on the initial quantity sintered into source, which was a few atomic percent, and was expected to be constant throughout the deposition.

The CdTe films were deposited at 550 °C substrate and 840 °C source temperatures in an N_2_ ambient onto 4″ × 4″ SCHOTT AF45 glass coated with a fluorinated tin oxide/tin oxide bilayer and CdS films. After growth, some films were annealed in an ampoule with Cd overpressure to place the group V dopants on Te sites. Similar activation was also achieved using Cd overpressure in a close-spaced sublimation chamber, which indicates the activation process can be engineered for manufacturing and ultimately integrated into a CdTe deposition sequence. This post growth activation can be necessary if incorporation alone is not sufficient to obtain high acceptor levels while retaining electron lifetime. Also, post-deposition annealing of the films, coupled with rapid cooling can eliminate excess dopant, reduce compensating defects and homogenize the intragranular dopant distribution.

**Figure 2:**
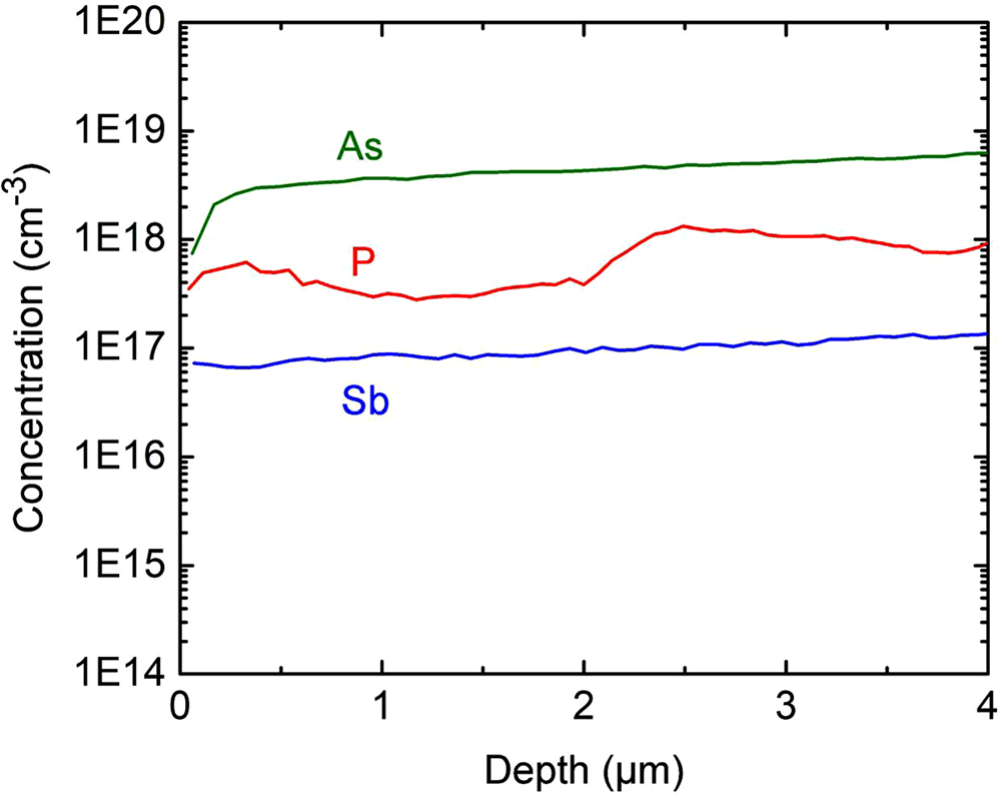
SIMS concentration versus depth for films with P, As and Sb dopants.

SIMS depth profile results of as-deposited films, from the exposed CdTe surface to the CdS interface, are shown in Fig. 2. The profiles show nearly uniform dopant incorporation at the targeted 10^17^−10^18^ atoms-cm^−3^ range, which is sufficient to achieve hole densities exceeding 10^16^ cm^−3^ throughout the film thickness. SIMS analysis was not performed on the annealed films, as the rapid annealing (4 minutes at 600 C) is not likely to result in significant diffusion/segregation of the group V dopants.

**Figure 3:**
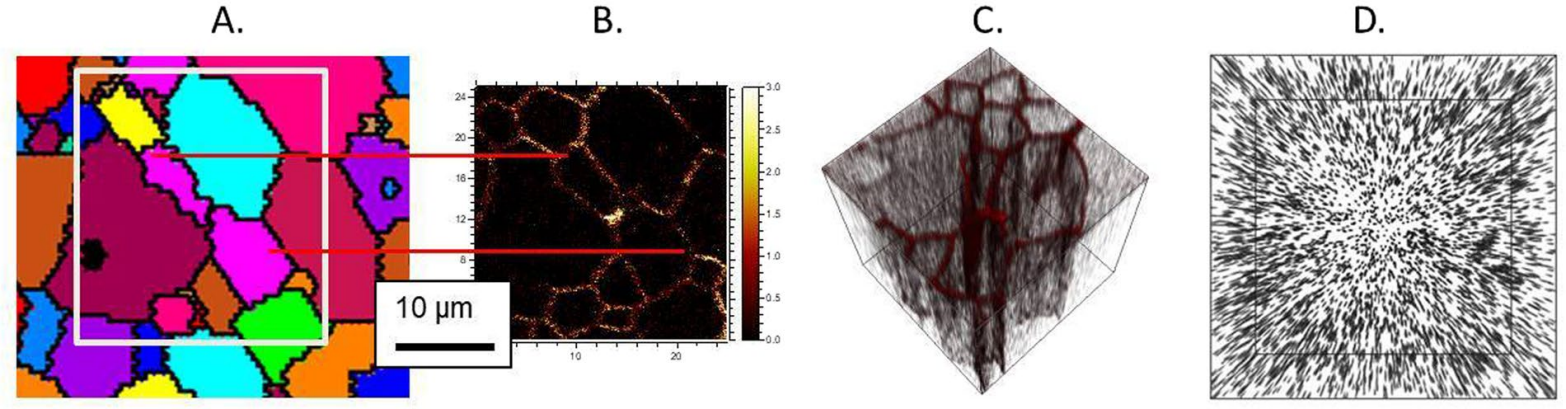
(**A**) EBSD grain orientation map and corresponding. (**B**) TOF-SIMS phosphorous image 25×25 µm showing phosphorous accumulates at the grain boundaries during *ex-situ* dopant incorporation in CdTe and (**C**) 3-D rendering of the phosphorous distribution (25 × 25 × 0.9 µm) for *ex-situ*-diffusion polycrystalline CdTe. This is contrasted with (**D**) a 3-D rendering of the phosphorus distribution (25 × 25 × 1.0 um) for *in-situ* incorporation by vapor transport deposition. The films have similar grain structure, but phosphorous grain boundary aggregation is not observed in the latter.

A critical component of *in-situ* doping is improving the ability to incorporate dopants uniformly throughout the film without grain boundary aggregation or other surface features. Figure 3 compares 3-dimensional time-of-flight secondary ion mass tomography on a sample grown by *in situ* incorporation contrasted with a sample grown by *ex-situ* diffusion. It is already well-established that Sb, As, and P diffuse orders of magnitude more quickly along grain boundaries than by slow substitutional bulk diffusion, and in the case of P, fast interstitial bulk diffusion^13,14,27^. These differences in diffusion rates lead to strong grain boundary aggregation and make it difficult to obtain uniform incorporation and precisely controlled material properties for device applications. On the other hand, Fig. 3D indicates that *in-situ* incorporation allows for controlled and uniform doping not just through the film depth but also laterally. Graded doping could be engineered if desired, which offers a new mechanism to control the properties of polycrystalline materials.

**Figure 4:**
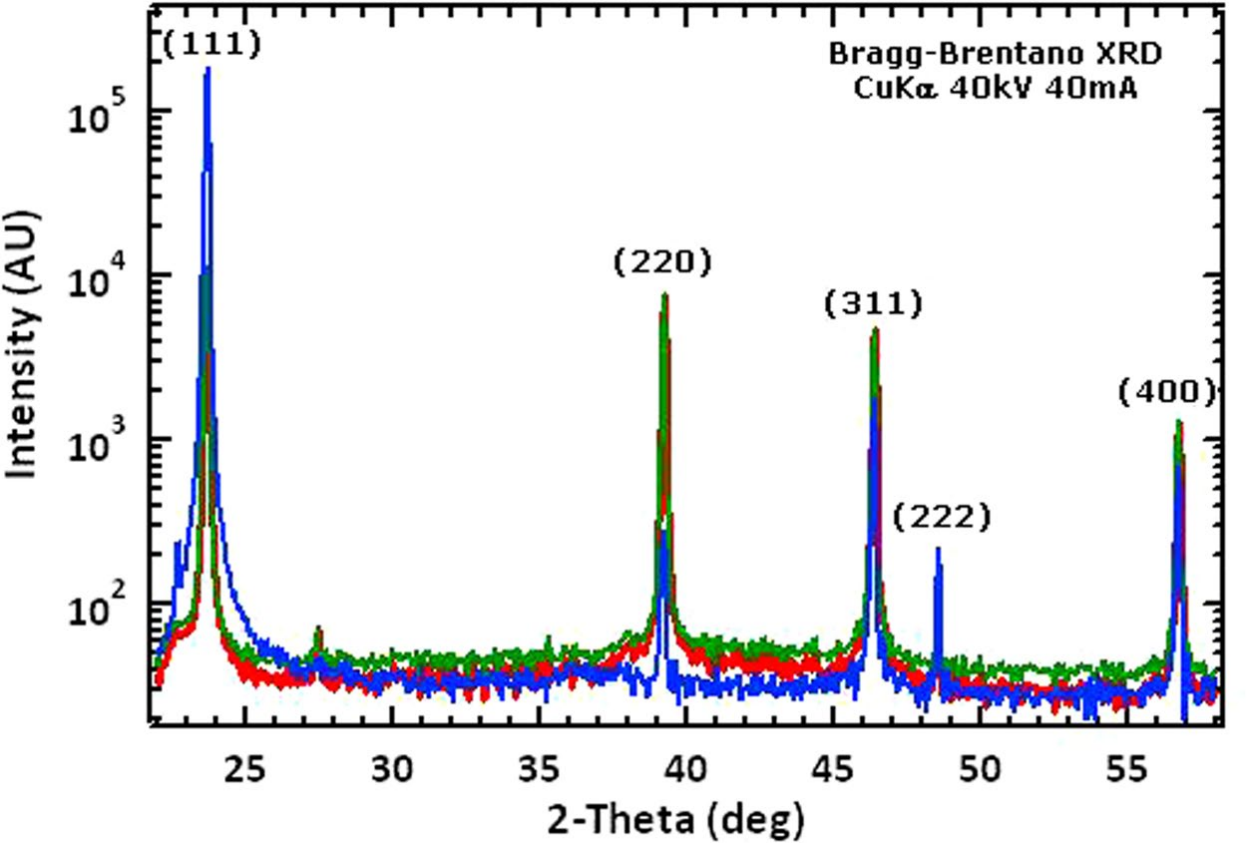
XRD patterns of CdTe films doped with P (red), As (green), and Sb (blue).

**Figure 5:**
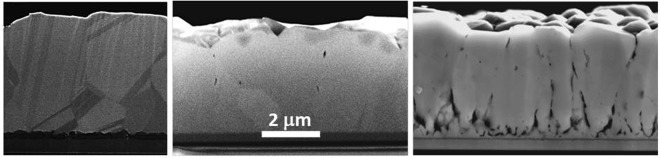
SEM cross sectional images of 5–6 micron thick CdTe films: Undoped (left) and doped with ~10^17^ cm^−3^ incorporated levels of Sb (middle) and >10^18^ cm^−3^ levels of As (right).

XRD patterns, shown in Fig. 4 with a logarithmic intensity axis, indicated that the doped films are single phase CdTe with zincblende structure. Films deposited with P and As exhibited near-random orientation to the substrate, but films deposited with Sb consistently exhibited strong (111) texture in which the (222) reflection at 2θ = 48.5 deg is also obtained. In all cases, the terminating CdTe grains were well-faceted and exhibited grain width-to-thickness aspect ratio varying from 0.2 to 0.8. Cross-section scanning electron microscope measurements (Fig. 5) show columnar film growth and varying porosity, depending on dopant levels and conditions. In general, it is possible to obtain high quality dense films as shown in Fig. 5 for Sb doping at incorporation levels of 10^17^ cm^−3^. However, attempting to incorporate too much dopant can create film quality issues. For example, Fig. 5 also illustrates an attempt to incorporate high levels of As, which results in films exhibiting significant porosity at the CdS-CdTe interface and along grain boundaries due to re-evaporation of secondary phases.

**Figure 6:**
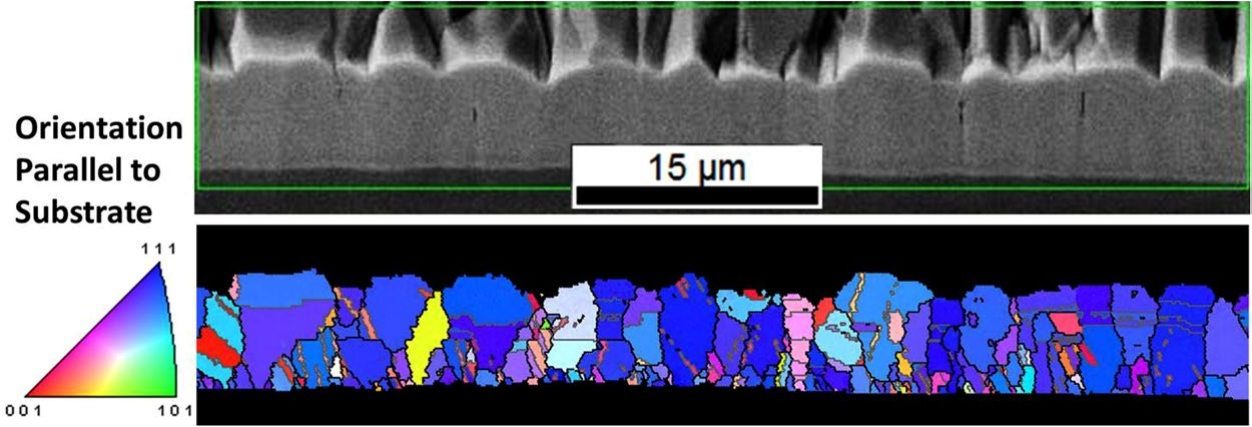
SEM image and EBSD inverse-pole-figure (IPF) map of the cross-section of Sb-doped CdTe film in which color coding is used to highlight crystallographic directions parallel normal to the substrate surface (blue for <111>), low angle (grey), and high angle (black) grain boundaries.

Cross-sectional analysis with EBSD provides further support of the crystallographic texture as shown in Fig. 6. Although the analysis was performed on the cross section, EBSD is able to investigate the crystallographic orientation in different directions. Figure 6 shows an IPF map, where the color is related to the crystallographic orientation normal to the sample surface. Referring to the orientation triangle, shown on the same figure, it is clear that the sample has (111) preferential orientation. In Fig. 6, dark lines are grain boundaries, and gray lines are coincidence site lattice Σ3 boundaries, which in most cases are twin boundaries.

The doped films exhibit positive lateral Seebeck voltage (described in the methods section) ranging from +3 to +10 mV, corresponding to a mean Seebeck coefficient of +25 mV/K, consistent with p-type conductivity. Comparatively, CdTe films deposited without dopants or oxygen yielded Seebeck voltages from 0 to −3 mV, suggesting intrinsic or slightly n-type conductivity. For these nominally un-doped films, positive voltages were only obtained after post-deposition annealing in CdCl_2_:O_2_ ambient at 400–450 °C for 10–30 mins.

**Figure 7:**
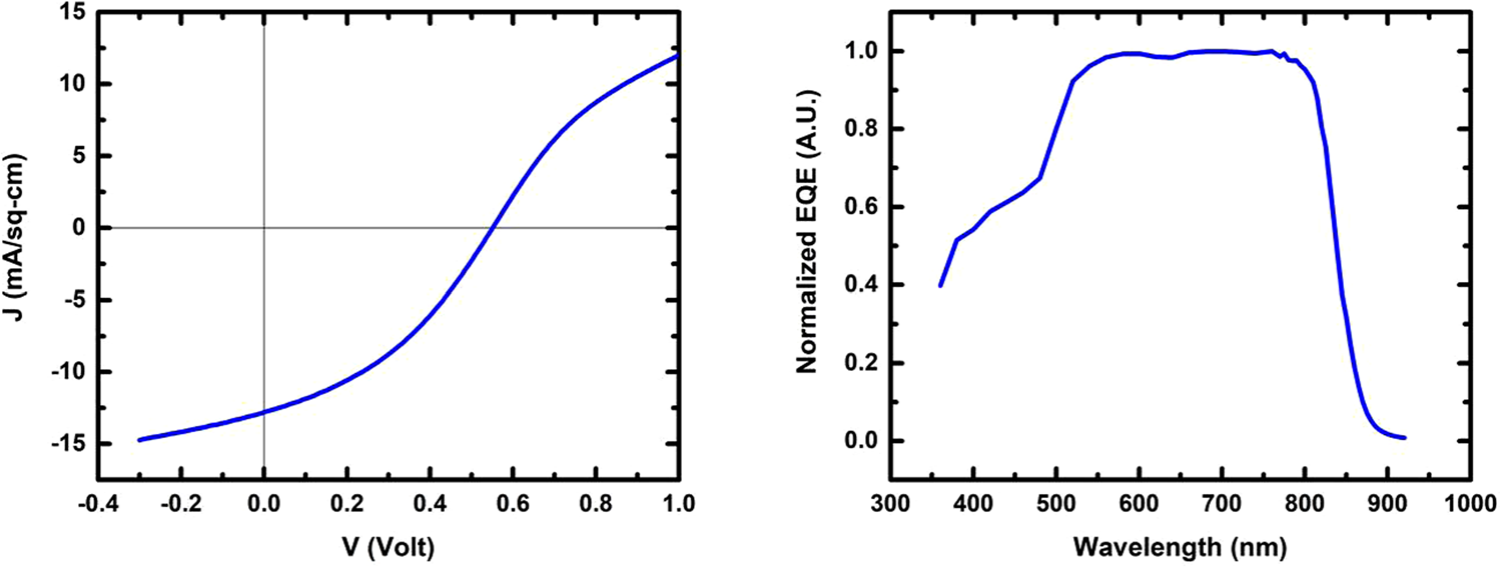
J-V behavior and EQE response at 0 V of diagnostic CdTe:Sb cell used to measure *N*_*CV*_.

To survey the effectiveness of the VTD approach for *in-situ* doping, the effort focused on achievable hole density with incorporation levels of the different Group V dopants, prior to adding the complexity of a CdCl_2_ treatment, Cu diffusion, and device optimization for such a variety of dopants and incorporation levels. Devices fabricated with graphite dot contacts were analyzed by current-voltage (J-V), capacitance-voltage (CV), and external quantum efficiency (EQE) methods. From these, values for open circuit voltage (*V*_*OC*_), short circuit current (*J*_*SC*_), and charge response (*N*_*CV*_) were obtained. The J-V and EQE response of a cell is indicated in Fig. 7, indicating p-type doping in the CdTe of a cell made with Sb doping, after activation with Cd vapor at 600 °C. A cell made with Sb doping at *N*_*CV*_ = 5 × 10^15^ cm^−3^, treated in CdCl_2_:O_2_ vapor, yielded *V*_*OC*_ = 714 mV and *J*_*SC*_ = 23.6 mA/cm^2^, suggests that future cell optimization paths can be consistent with present CdTe module fabrication methods.

**Figure 8:**
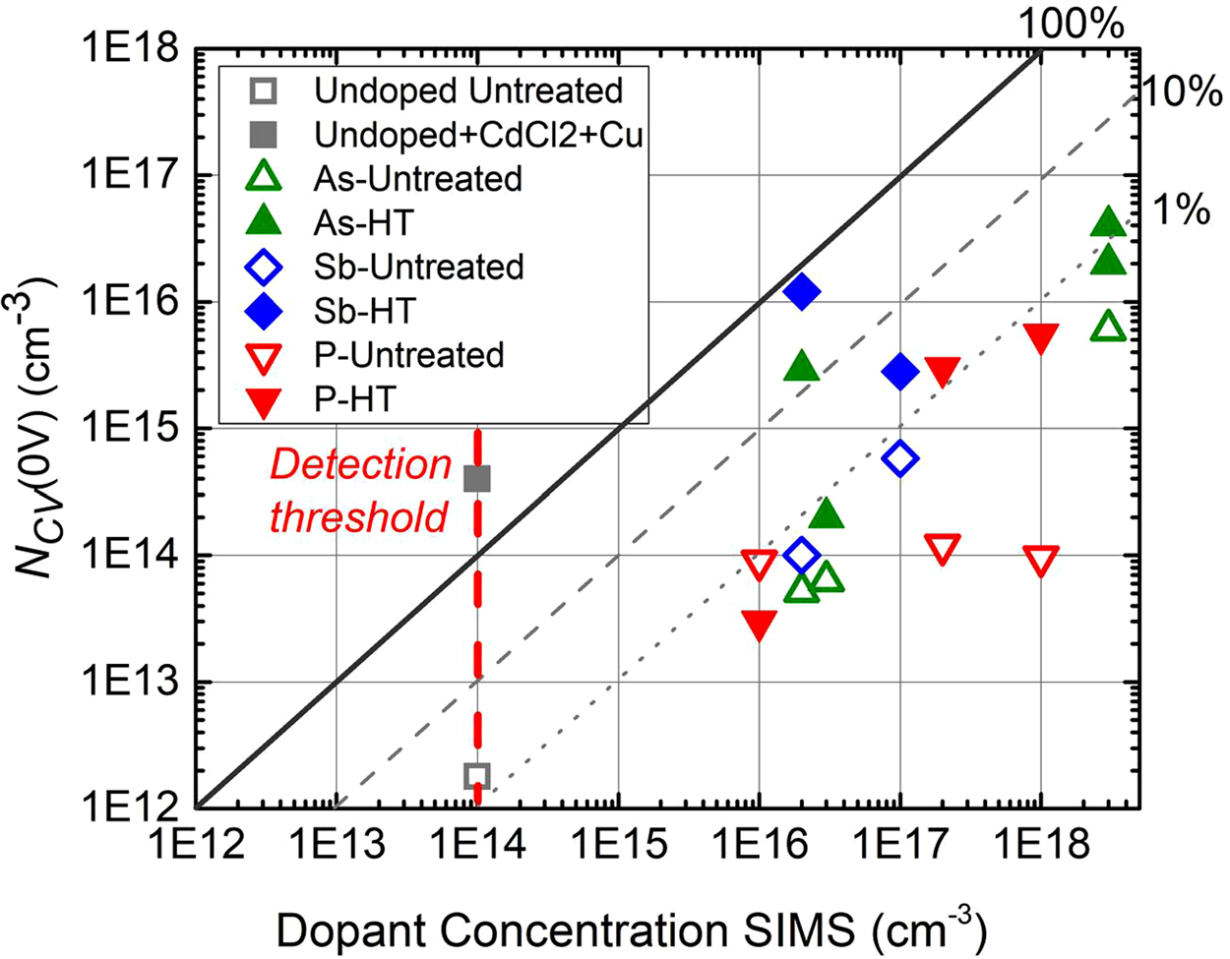
N_CV_ at zero volts versus SIMS concentration. The red dotted line indicates the SIMS detection threshold for the Group V dopants and used as a reference for un-doped CdTe. The square symbols indicate the range of raw and fully optimized CdTe films using CdCl_2_:O_2_ treatment and diffused Cu contact.

Capacitance-voltage measurements were utilized to determine hole density, *N*_*CV*_, as a function of dopant incorporation levels determined by SIMS, as described in the methods section. Figure 8 summarizes the results for as-deposited and activated CdTe films with different dopants. Cells made with As and Sb-doped CdTe exhibit *N*_*CV*_ exceeding 10^16^ cm^−3^.

To understand this in terms of the incorporated dopant concentration, Table 2 summarizes the mean SIMS dopant concentration, measured free charge response at 0 V DC bias, *N*_*CV*_(0 V) and TRPL lifetime for VTD films that yielded that highest *N*_*CV*_ values, compared to an IEC baseline cell using CdTe deposited in oxygen, and completed with a CdCl_2_ treatment and Cu-diffusion step prior to contacting. Figure 8 shows the obtained free charge response at zero volts, *N*_*CV*_(0 V), versus the SIMS dopant concentration in a wider sample set before and after activation treatment. All Group-V doped films exhibit higher *N*_*CV*_ levels than un-doped films. The activation treatment consistently increased the doping concentration by >10X compared to that in the raw films.

**Table 2:** Dopant concentration by SIMS, doping density, doping efficiency, and TRPL lifetimes.

Group V Dopant	Activation	Dopant Concentration SIMS (cm^−3^)	N_A_ (cm^−3^)	Doping Efficiency (%)	τ_1_/τ_2_ TRPL (ns/ns)
P	None	2.0 × 10^17^	8.0 × 10^13^	0.06	0.17/1.4
P	Cd vapor		1.0 × 10^15^	1.5	
As	None	3.0 × 10^18^	6.0 × 10^15^	0.2	0.10/1.5
As	Cd vapor		3.7 × 10^16^	1.2	
Sb	None	1.0 × 10^17^	4.0 × 10^13^	0.2	0.45/1.6
Sb	Cd vapor		1.9 × 10^16^	19	

In Table 2, the cell made with *as-deposited* un-doped CdTe, corresponding to the first row, exhibited the lowest free charge response *N*_*CV*_(0 V). The cell made with *annealed* un-doped CdTe, treated at 550 °C, yielded similar *N*_*CV*_(0 V) levels as a 15% efficient “baseline” cell processed with un-doped CdTe and treated with O, Cl and Cu during cell processing, shown in the last row. The *as-deposited* group V doped films yielded cells with elevated *N*_*CV*_ prior to activation, compared to un-doped. After activation, *N*_*CV*_ increased ~10X, with the highest, As-doped, reaching >10^16^ cm^−3^. Based on the SIMS incorporation levels and minimum *N*_*CV*_ values, doping activation efficiencies of 1.5%, 1.2%, and 19% were obtained for P, As and Sb, respectively.

The processing window for activation was explored by varying the Cd partial pressure and activation temperature. Table 3 shows the resulting *N*_*CV*_ values obtained for As-doped films, where the film activation temperature was evaluated from 500 °C to 625 °C at Cd partial pressures from 1 to 35 Torr. *N*_*CV*_ values from mid-10^15^ cm^−3^ to low-10^16^ cm^−3^ were obtained at 550 °C and 600 °C independent of the Cd partial pressure.

**Table 3:** Doping density (cm^−3^) of CdTe:As films after ampoule activation with Cd overpressure.

	T film →	500 °C	550 °C	600 °C	625 °C
T_Cd_	P^sat^_Cd_				
390 °C	1 Torr	4.4 × 10^15^	2.0 × 10^16^	3.4 × 10^16^	X
500 °C	13 Torr	3.6 × 10^16^	2.0 × 10^16^	3.7 × 10^16^	X
550 °C	35 Torr	X	9.0 × 10^15^	1.5 × 10^16^	3.8 × 10^15^

Effective electron lifetime was measured by 1-photon excitation time-correlated single photon counting to generate time-resolved photoluminescence curves. Laser pulses with a wavelength of 640 nm were fired at a rate of 1.1 MHz through the glass onto the CdTe depletion region and fit with bi-exponential decay curves. The measured TRPL lifetime, shown in the last column of Table 2, is lower in doped, un-treated films than in 15% efficient IEC “baseline” VTD CdTe/CdS cells. Without the passivation obtained by O_2_ during CdTe growth, CdCl_2_ post treatment in air, and Cu diffusion from the back contact, effective lifetime will be dominated by high surface and grain boundary recombination velocity^33–36^. The CdTe film in a typical superstrate CdTe/CdS cell exhibit raw τ_1_ ~ 0.6 ns and τ_2_ from 2 to 5 ns. In the present study, cells made with as-deposited doped CdTe grown without CdCl_2_ treatment have τ_1_ ~ 0.5 ns and τ_2_ between 1.2 and 1.6 ns. While lower than the baseline, these values are still adequate for reasonable solar cell performance and are very good for cells that have not received CdCl_2_ treatment^37^. These results indicate that Sb, As, or P dopants incorporated at concentrations approaching 10^17^ cm^−3^ do not severely limit electron lifetime in the CdTe film. Prospects for enhancing device V_OC_ through increased doping are indicated by results for Sb in which cells having acceptor levels of 7–9 × 10^15^ cm^−3^ yielded devices with V_OC_ > 700 mV with only CdCl_2_ treatment and with no copper processing. This is substantially higher V_OC_ than obtained with un-doped CdTe devices, which have acceptor levels of 4 × 10^14^ cm^−3^ and V_OC_ < 500 mV. In the future, effective lifetime and V_OC_ improvements are anticipated by modifying post-deposition activation processes.

**Figure 9:**
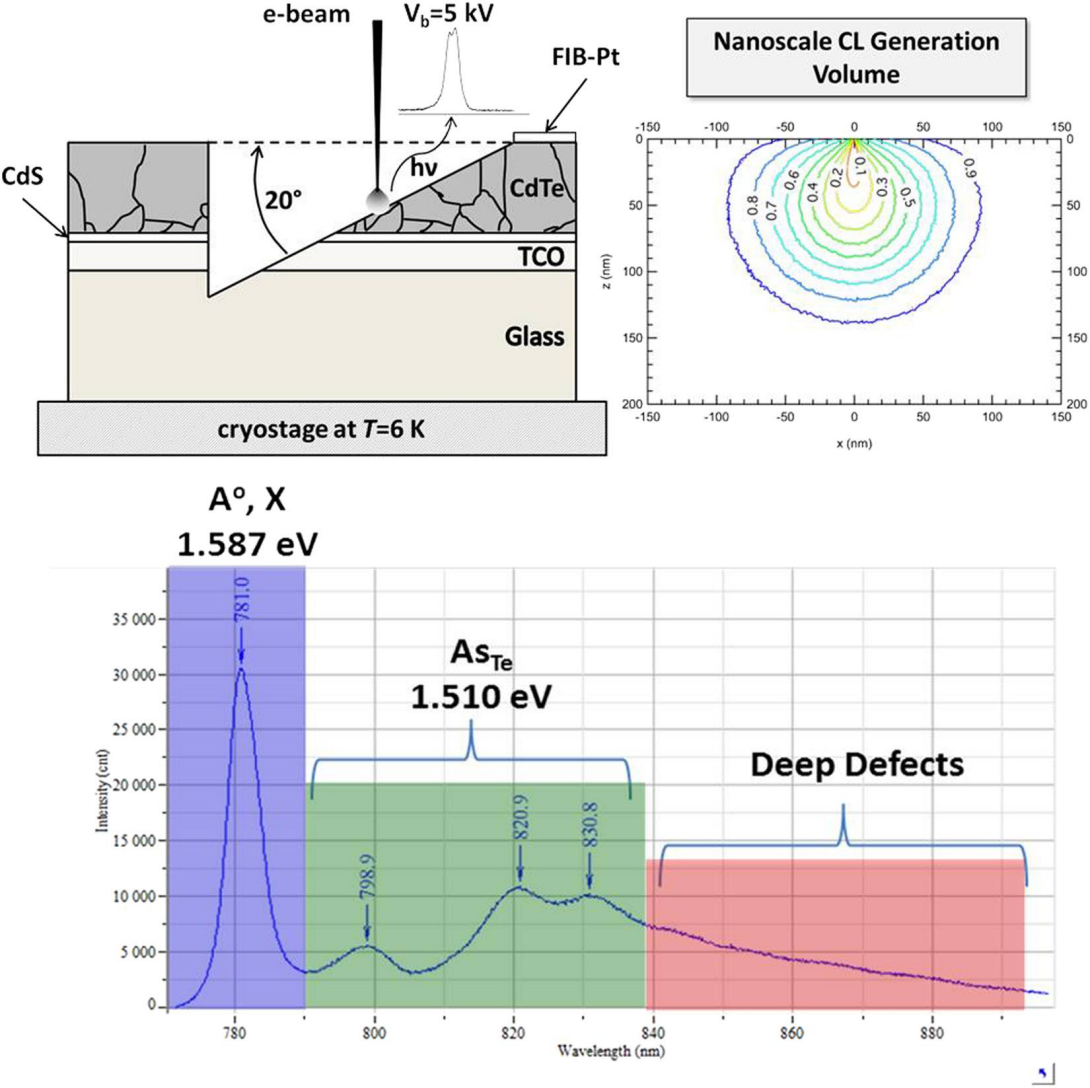
CL sample preparation and generation volume (top) and CL spectrum (6 K) for CdTe:As (bottom).

**Figure 10:**
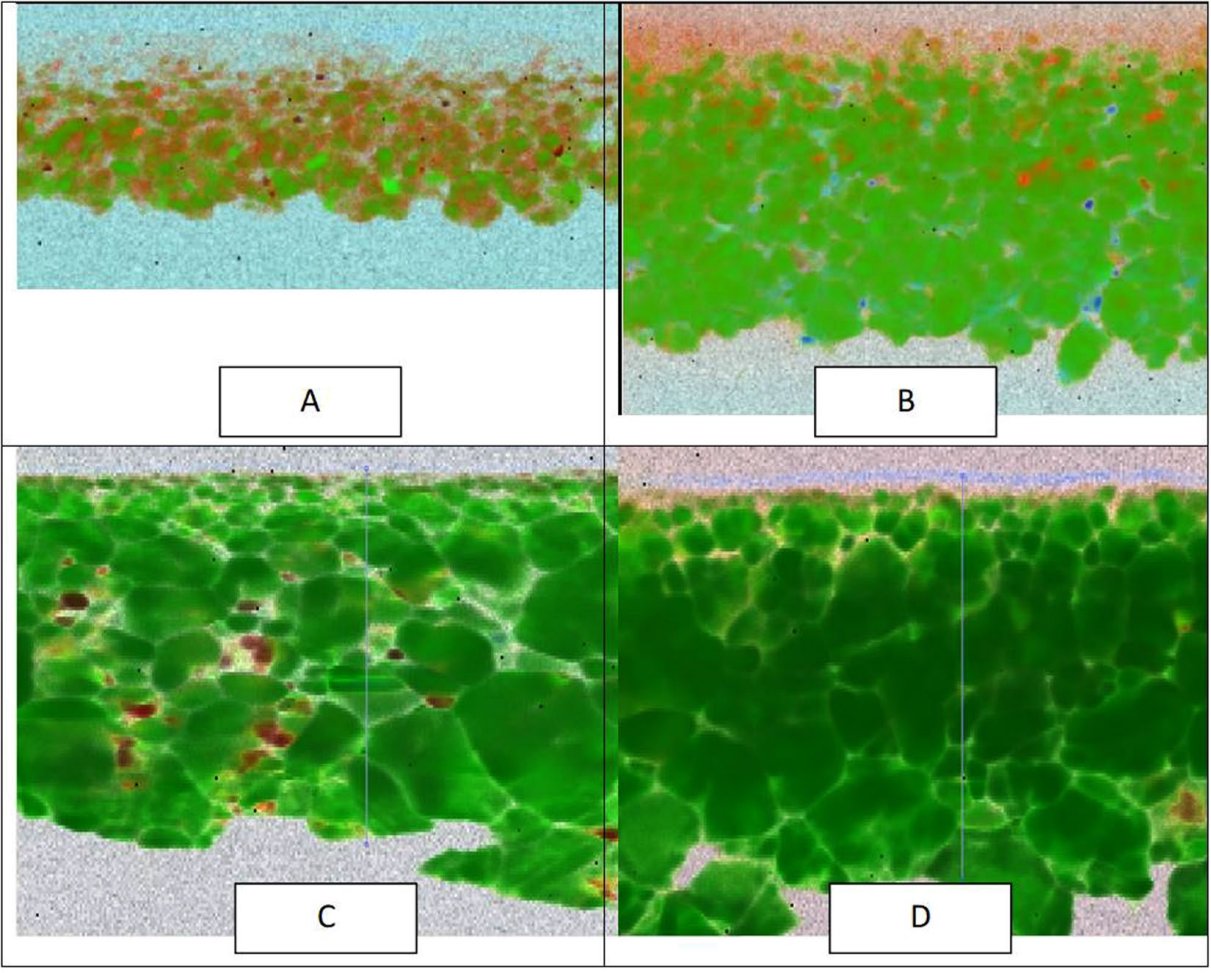
Beveled cross-section CL maps of CdTe:As and CdTe:Sb films: (**A**) CdTe:As prior to activation; (**B**) CdTe:As after activation; (**C**) CdTe:Sb prior to activation; (**D**) CdTe:Sb after activation.

Validation of the formation of substitutional defects was obtained using cathodoluminescence (CL) spectroscopy of beveled cross-sections of As-doped and Sb-doped cells, before and after Cd overpressure anneals intended to place As or Sb onto Te sites. Figure 9 shows the sample preparation geometry and generation volume used to obtain CL imaging maps through doped films before and after activation annealing. The spectrum of the As annealed sample is shown in Fig. 9, and for the purposes of mapping in which CL spectra are taken at every point in the image, and assigned the colors blue, green and red for excitonic, As_Te_, and deep defect peaks, respectively. A similar process was used for excitonic, a shallow Sb acceptor, and deep defect emission for Sb doping using ref.^38^. In Figs 10A,B the green throughout the grains in the images illustrate that we have indeed achieved uniform intra-grain substitutional As_Te_ doping throughout the film before and after activation. Further, and consistent with CV data, the Cd vapor anneal increases the activation of As on Te throughout the film, with the annealed film exhibiting 80% of the luminescence arising from the substitutional defects, compared to the raw film which exhibits equal luminescence from substitutional and deep defects. For Sb-doped films, shown in Figs 10C,D, the fraction of substitutional defects is even higher than obtained with As.

For Sb-doped films, the images also show the prevalence of an acceptor peak at approximately 57 meV throughout the film. Interestingly, theoretical calculations have long predicted that the transitional energy levels of GrV elements on Te sites would progressively become deeper with atomic size, so whereas As and P are predicted and observed to have transition energies less than100 meV^20,21^, Sb is predicted to be 230 meV deep^20^. As a result, Sb has often been considered a less attractive dopant that would yield relatively poor activation ratios to As or P. However, here we observe an Sb acceptor transition at nearly the same level as As, consistent with other CdTe:Sb luminescence data^38,39^. While it is possible that the shallow Sb acceptor is caused by a complex, the emergence of the well known As_Te_ site from parallel processing suggests this is a Sb substitutional site at a much shallower level than earlier theoretical predictions. A shallow transition energy combined with an atomic radius similar to Te makes Sb a far more favorable dopant than previously considered, as manifested by quality films and high activation ratio and hole density here.

## Conclusions

Longstanding carrier concentration limitations in polycrystalline CdTe films are overcome by incorporating group V elements during growth, followed by a short post-growth anneal in Cd vapor and fast cooling to form substitutional V_Te_ acceptors. SIMS depth profiles indicate sufficient P, As, and Sb incorporation levels, between 10^17^ cm^−3^and 10^18^ atoms-cm^−3^, are attained by vapor mixing from compounds containing the group V elements within the CdTe source in Cd-rich ambient. As and Sb enable acceptor concentrations >10^16^ cm^−3^ without compromising carrier lifetime. CL clearly images the desired dopant throughout polycrystalline films for the first time, tracks the enhancement of Group V atoms on Te sites by Cd overpressure anneals, and demonstrates lateral and depth doping uniformity. The emergence of a shallow Sb acceptor and high activation ratios make Sb an attractive dopant for future work. Carefully controlled *in-situ* doping and anneals provide new paths for controlling carrier concentration in polycrystalline thin films by orders of magnitude to advance both low cost solar energy and other thin film semiconductor applications.

## Methods

The dopant compounds were synthesized in quartz ampoules using 6 N purity elements, fired for 10 days at 650 °C, followed by vapor transport as a final purification step.

CdS was deposited by chemical surface deposition to a thickness of ~100 nm^40^. The CdS then received a CdCl_2_ vapor treatment at 415 °C for 15 minutes to crystallize and dehydrate the CdS film. Upon completion of the CdTe deposition, the films were rapidly cooled by injection of N_2_ during a high-speed translation away from the deposition zone, resulting in an initial 200 °C temperature drop at a cooling rate of >5 °C/s. CdTe film thicknesses of 5–7 microns were routinely achieved.

Post-deposition annealing was carried out in sealed quartz ampoules on 1 cm × 1 cm coupons taken from the CdTe-coated plates. The annealing was performed in a horizontal furnace with measured temperature profile, at 550 °C–600 °C for 4 minutes. For this, the CdTe coupons were sealed in evacuated quartz ampoules, at less than 1 × 10^−6^ Torr, together with a 300 mg lump of Cd metal, to provide a Cd vapor excess and a 2 mm^3^ chunk of graphite, to getter residual oxygen. The location of the coupons and the Cd lump was varied to control the annealing temperature and the Cd partial pressure.

Solar cells were fabricated as diagnostic devices by performing a light viscous surface etch in ethylenediamine (EDA) to remove residual surface oxides and create an ultra-thin, <10 nm, Te surface. This process was followed by rinsing and then applying a graphite dot contact with Acheson 505SS high purity ink, which was air dried at 30 °C for 30 minutes.

To evaluate film phase composition, x-ray diffraction (XRD) was measured by a Rigaku d/Max diffractometer in both Bragg-Brentano and glancing incidence modes at 1 degree incidence using Cu-kα radiation. Grain size and film porosity were assessed by high-resolution optical microscopy and scanning electron microscopy. For the electron back scattered diffraction (EBSD) measurements we used an EDAX Hikari detector installed in an FEI field-emission scanning electron microscope. For the analysis, the sample was tilted by 70°. To avoid shading effects, due to surface roughness, the sample was ion milled before analysis in a JEOL cross-section polisher using Ar^+^ ions. For EBSD measurements on the surface, the sample was ion milled at 4 kV for 15 minutes. For EBSD measurements on the cross section, the sample was ion milled at 4 kV for 12 hours.

The film conductivity type was evaluated by the thermal voltage generated between a heated spring-loaded steel probe and a room temperature probe. A spring-loaded probe heated to 225 °C was gently lowered onto the CdTe film in conjunction with a room temperature probe at a distance of 1 cm.

**Figure 11:**
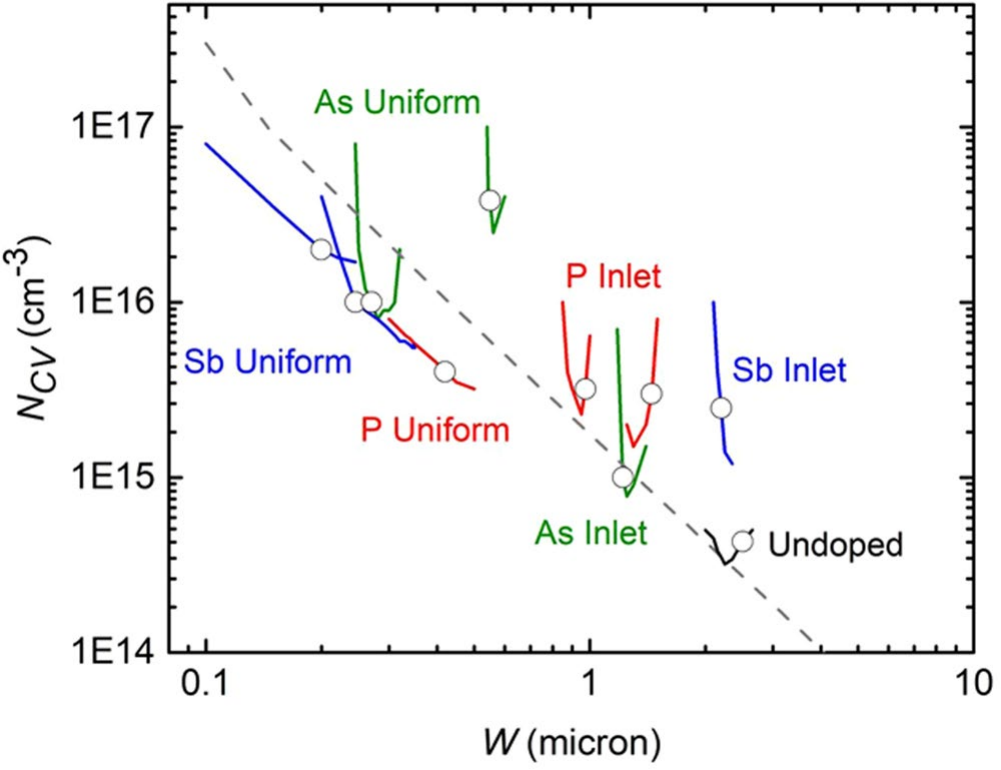
Mott-Schottky plot at 10 kHz and 300 K N_CV_ versus W obtained for cells made with different Group V dopants using inlet and uniform dopant delivery methods, compared with typical results using un-doped CdTe.

J-V measurements were made in the dark and under AM1.5 global simulated light using an Oriel simulator and Keithley 2400 SMU. The voltage was typically swept from −0.5 V to +1 V in ~7 mV steps. CV measurements were carried out using an Agilent 4284 A LCR meter. DC voltage bias was provided using a Keithley 2400 SMU and the Agilent 16065 C external bias adapter. For CV measurements, we use a fixed AC voltage signal and varying DC voltage bias, measuring capacitance as a function of VDC and, since the depletion width is varied with VDC, as a function of *W*. We calculated the responding charge density, *N*_*CV*_, from:

3 $$N{(W)}_{CV}=-\,\frac{{C}^{3}}{q{\epsilon }{A}^{2}(\frac{dC}{dV})}=-\,\frac{2}{q{\epsilon }{A}^{2}}{[\frac{d(1/{C}^{2})}{dV}]}^{-1}$$

where *W* is the depletion width, *A* is device area, *q* is electron charge, and *ε* = 13.5*ε*_0_. At this time, ascribing the measured charge response to acceptors is based on the *N*_*CV*_ versus *W* behavior compared with an ideal abrupt junction. Shown in Fig 11 is a compilation plot of *N*_*CV*_ versus W with *N*_*CV*_ at zero volts indicated by an open circle on each data set. The data, for cells after dopant activation, are plotted along with a dotted line indicating the expected behavior for a device with uniform doping and abrupt junction. Each sample exhibits the characteristic U-shape found with CdTe thin film solar cells^41^, with the zero volt charge response falling near the U-minimum in all but one case. The data shows higher doping levels obtained with uniform dopant distribution in the CdTe source, enabling higher dopant incorporation in the deposuted films.

Secondary Ion Mass Spectrometry (SIMS) is a powerful analytical technique for determining elemental and isotopic distributions in solids^42,43^. Dopant incorporation levels in CdTe films were measured by SIMS depth profiling at the Evans Analytical Group. The elements Sn and Te were used as markers for the extent of the CdTe film and beginning of the tin oxide window layer stack. The detection limit for dopants P, As and Sb were ~ mid 10^14^ atoms-cm^−3^. Time of Flight SIMS depth profiling (TOF SIMS) and 3-D tomography was utilized to measure the diffusion profiles and lateral distribution of dopants in CdTe in this study with an ION-TOF TOF-SIMS V spectrometer at NREL. A Cs+ or oxygen ion beam with an energy of 3 KeV was utilized as the sputtering beam (sputtering current 25 nA). The sputtering ion beam was scanned over an area of 250 × 250 µm. Secondary ions for analysis were created by a 3-lens 30 keV BiMn ion gun. Two different measurement modes were utilized in this study; depth-profiles were collected with a high data-density to probe the dopant concentration through the film thickness. In this case a Bi^+^ beam was utilized (operated in bunched mode; 11 ns pulse width, analysis current 1 pA), scanned over a 50 micron area. 3-D tomography was completed with 100 nm lateral resolution using a Bi^3++^ primary ion-beam cluster (100 ns pulse width, 0.1 pA pulsed beam current), a 25 × 25 µm area was sampled with a 256:256 primary beam raster, utilizing a primary ion beam-dosage of 1 × 10^13^ ions/cm^2^ for each imaging cycle. After completion of the SIMS measurements the depth of the craters was determined by optical interference light microscopy, in order to convert the SIMS sputter time scale to a sputter depth scale.

Effective lifetime was measured by 1 photon excitation time-resolved photoluminescence (TRPL). Laser pulses with a wavelength of 640 nm were fired at a rate of 1.1 MHz through the glass onto the CdTe depletion region. Photoluminescence was collected and separated from scattered light with a 840-nm bandpass filter and Si avalanche photodiode detector. Decay curves were generated using time-correlated singe photon counting and fit by a biexponential function, where τ_1_ and τ_2_ describe the exponential decay rates at the initial and final sections of the curve, respectively^37^.

For two dopants, As and Sb, cathodoluminescence (CL) was conducted on beveled surfaces, prepared with a focused ion beam-scanning electron microscope (FIB-SEM). An initial 20°-wedge was milled through the film using a 30-kV ion beam, and then a 5-kV beam prepared the final surface for CL analysis. CL spectrum imaging was performed on a JEOL 7600 F Field Emission SEM equipped with a Horiba H-CLUE CL system. Samples were cooled to 6 K on a Gatan CF302 liquid-helium cold stage during CL at electron beam conditions of 5 kV and 4 nA.

## Data Availability

The datasets used to generate the figures and tables in the current study are available from the corresponding author on reasonable request.
